# Choosing between medical management and liver transplant in urea cycle disorders: A conceptual framework for parental treatment decision‐making in rare disease

**DOI:** 10.1002/jimd.12209

**Published:** 2020-01-13

**Authors:** Maya T. Gerstein, Anne R. Markus, Kan Z. Gianattasio, Cynthia Le Mons, Janice Bartos, David M. Stevens, Nicholas Ah Mew

**Affiliations:** ^1^ Department of Health Policy and Management, Milken Institute School of Public Health The George Washington University Washington District of Columbia; ^2^ National Urea Cycle Disorders Foundation Pasadena California; ^3^ Rare Disease Institute, Children's National Health System Washington District of Columbia

**Keywords:** decision‐making, liver transplant, qualitative research, treatment choice, urea cycle disorders

## Abstract

Urea cycle disorders (UCD) are rare inherited metabolic disorders caused by deficiencies of enzymes and transporters required to convert neurotoxic ammonia into urea. These deficiencies cause elevated blood ammonia, which if untreated may result in death, but even with optimal medical management, often results in recurrent brain damage. There are two major treatments for UCD: medical management or liver transplantation. Both are associated with mortality and morbidity but the evidence comparing outcomes is sparse. Thus, families face a dilemma: should their child be managed medically, or should they undergo a liver transplant? To (a) describe the factors that contribute to treatment choice among parents of children diagnosed with UCD and to (b) organise these factors into a conceptual framework that reflects how these issues interrelate to shape the decision‐making experience of this population. Utilising grounded theory, qualitative data were collected through semi‐structured interviews with parents (N = 35) and providers (N = 26) of children diagnosed with UCD and parent focus groups (N = 19). Thematic content analysis and selective and axial coding were applied. The framework highlights the life‐cycle catalysts that frame families' personal perceptions of risks and benefits and describes the clinical, personal, social, and system factors that drive treatment choice including disease severity, stability, and burden, independence, peer experiences, and cost, coverage and access to quality care. Findings equip providers with evidence upon which to prepare for productive patient interactions about treatment options. They also provide a foundation for the development of patient‐centred outcome measures to better evaluate effectiveness of treatments in this population.

## 1 INTRODUCTION

Urea cycle disorders (UCD) are rare inherited disorders of metabolism caused by deficiencies of one of six enzymes and two transporters required for ammonia detoxification and urea synthesis. Disruption of the urea cycle can result in hyperammonaemia which precipitates cytotoxic brain oedema and may result in death. In those who recover from acute hyperammonaemia, intellectual and developmental disabilities are common.^1^, ^2^, ^3^, ^4^, ^5^, ^6^

Despite significant improvements in medical management (MM) following the wider availability of alternative pathway medications, most individuals with UCD remain at high risk of hyperammonaemia.^7^, ^8^ Thus, an increasing number of patients have been undergoing liver transplantation (LT) as a procedure that “cures” the hyperammonaemia.^9^, ^10^, ^11^, ^12^, ^13^ However, LT is a complicated surgical procedure, which carries risk of mortality and morbidity and requires a life‐long regimen of immunosuppression.

Most patients with UCD are at elevated risk for disability or death at various times in their lives.^14^ This risk, although always present, is not easily quantified, especially among mild‐moderately affected patients. This ambiguity can make treatment decisions, like if and/or when to pursue LT, particularly challenging for patients. In other medical conditions, the decision to perform transplantation is often made because organ function has failed. However, for patients with UCD, the decision can be more complex as outcomes from medical therapy vary widely and because transplantation is ideally initiated when patients are stable rather than critically ill. There is also limited empirical evidence to support clinical guidance on treatment alternatives for patients with UCD, introducing ambiguity and personal judgement to the process of making treatment decisions. Clinicians involved in the diagnosis and treatment of UCD have long relied on non‐comparative research findings in combination with expert opinion to guide clinical practice and treatment counselling for these disorders.^15^ Thus, they have been unable to help patients and families weigh available treatment alternatives against key health outcomes like survival, neurocognitive status, and quality of life.^3^, ^6^, ^16^, ^17^, ^18^, ^19^, ^20^ A more detailed description of currently available treatment guidance and evidence to support treatment decision making in UCD is available in the appendix (Table A1).

Despite its complexity, no research has been conducted on how families of UCD patients make treatment choices in the absence of definitive evidence and clinical guidance, and the issues that influence their decision to pursue one option over another. This article describes the findings from an adapted grounded theory study that combines qualitative interview and focus group data to examine the decision‐making experience of families affected by UCD to (a) identify key factors families consider in evaluating and reaching a treatment choice and (b) build a conceptual framework that explores how these factors interrelate to drive treatment decision making in this population.

## 2 METHODS

### 2.1 Approach

This study utilised an adapted grounded theory approach, borrowing from Strauss and Corbin's systematic procedures for grounded theory.^21^

### 2.2 Data sources and collection

Qualitative data were collected from parents of children affected by UCD (N = 35) and their clinical providers (N = 26) through semi‐structured phone interviews lasting 45 to 90 minutes. Two in‐person parent focus groups (N = 19), lasting 90 minutes each, were conducted to validate interview findings. Interviews and focus groups were recorded and transcribed verbatim for use in analysis.

Interview guides were developed by integrating findings from a limited relevant evidence base on parent health care decision‐making in paediatric illnesses where transplant is offered as a treatment.^22^, ^23^, ^24^, ^25^, ^26^, ^27^, ^28^, ^29^, ^30^, ^31^, ^32^, ^33^, ^34^, ^35^, ^36^, ^37^ Guides were augmented with additional information from key informants including patient advocates and metabolic physicians. They were revised in response to patient review.

### 2.3 Sampling

Stratified purposeful sampling was used to recruit an initial pool of parent participants whose children were born in the United States after 1996 (ie, post‐FDA approval of alternative pathway medications) and diagnosed with one of four UCDs (argininosuccinate lyase deficiency [ALD], argininosuccinate synthetase deficiency [ASD], carbamylphosphate synthetase deficiency [CPSI], and ornithine transcarbamylase deficiency [OTC]) for which LT is a consideration. Parent recruitment was conducted through the National Urea Cycle Disorders Foundation (NUCDF) via listserv, social media, discussions boards, and one‐on‐one outreach. Parent participants varied in terms of their child's (a) disease severity and (b) treatment course at the time of recruitment. Stratified sampling was also utilised to recruit a national cross‐section of UCD providers through the Urea Cycle Disorders Consortium (UCDC) listserv and via one‐on‐one outreach, reflecting variation in location and provider type. After an initial round of recruitment, subsequent study participants were selected through theoretical sampling. Characteristics of interview and focus group participants are summarised in Tables 1 and 2. Limitations of our study sample are discussed in the appendix (Table A2).

**Table 1:** Characteristics of urea cycle disorder (UCD) parent interview and focus group participants

		Interviews (N = 35)	Focus groups (N = 19)	Total (N = 54)
Gender‐caretaker	Male	11% (4)	21% (4)	15% (8)
	Female	89% (31)	79% (15)	85% (46)
Sex‐child	Male	53% (19)	63% (12)	56% (30)
	Female	47% (16)	37% (7)	44% (24)
Age‐caretaker^a^	21‐29	9% (3)	6% (1)	7% (4)
	30‐39	54% (19)	44% (8)	51% (27)
	40‐49	23% (8)	22% (4)	23% (12)
	50+	14% (5)	28% (5)	19% (10)
Age‐child	0–1	3% (1)	11% (2)	6% (3)
	2‐5	31% (11)	31% (6)	31% (17)
	6‐11	34% (12)	11% (2)	26% (14)
	12‐18	23% (8)	36% (7)	28% (15)
	>18	9% (3)	11% (2)	9% (5)
Disease severity‐child	Neonatal onset	71% (25)	68% (13)	70% (38)
	Late onset	29% (10)	32% (6)	30% (16)
Treatment status‐child	Medical management	40% (14)	42% (8)	41% (22)
	Liver transplant	60% (21)	58% (11)	59% (32)
Age at transplant‐child (if applicable)^b^	0–1	68% (14)	55% (6)	62% (20)
	2–5	16% (3)	18% (2)	16% (5)
	6–11	11% (2)	18% (2)	13% (4)
	12–18	0% (0)	0% (0)	0% (0)
	>18	5% (1)	9% (1)	6% (2)
Race‐caretaker	White	91% (32)	95% (18)	92% (50)
	Black	6% (2)	0% (0)	4% (2)
	Other	3% (1)	5% (1)	4% (2)
Hispanic or Latino‐caretaker	Yes	9% (3)	5% (1)	7% (4)
	No	91% (32)	95% (18)	93% (50)
Highest level of education‐caretaker^a^	Less than high school degree	0% (0)	0% (0)	0% (0)
	High school degree	3% (1)	0% (0)	2% (1)
	Some college	11% (4)	11% (2)	11% (6)
	Associate degree	6% (2)	6% (1)	6% (3)
	Bachelor's degree	40% (14)	39% (7)	40% (21)
	Graduate degree	40% (14)	44% (8)	41% (22)
Employment status caretaker^a^	Employed, part‐time	34% (12)	28% (5)	32% (17)
	Employed, full‐time	37% (13)	50% (9)	41% (22)
	Not employed	23% (8)	17% (3)	21% (11)
	Retired	3% (1)	0% (0)	2% (1)
	Disabled, not able to work	3% (1)	5% (1)	4% (2)
Household income^a^	<$25 000	9% (3)	6% (1)	8% (4)
	$25 000–$49 999	8% (3)	6% (1)	7% (4)
	$50 000–$74 999	3% (1)	11% (2)	6% (3)
	$75 000–$99 999	17% (6)	22% (4)	19% (10)
	$100 000‐$149 000	40% (14)	43% (8)	41% (22)
	$150 000–$200 000	14% (5)	6% (1)	11% (6)
	> $200 000	9% (3)	6% (1)	8% (4)

a n = 18 for focus group characteristic; n=53 total; 1 focus group participants failed to respond to all questions.

b n = 20 for interviews; n = 11 for focus groups; n = 31 total.

**Table 2:** Characteristics of urea cycle disorder (UCD) provider interview participants (N = 26)

Gender	Male	34% (9)
	Female	66% (17)
Age	31‐36	23% (6)
	37‐42	23% (6)
	43‐48	15% (4)
	49‐54	15% (4)
	55‐60	8% (2)
	>60	16% (4)
Clinical degree	MD	76% (20)
	Registered Nurse/Nurse Practitioner	8% (2)
	Genetic counselling	12% (3)
	Nutrition	4% (1)
Race	White	88% (23)
	Black	0% (0)
	Other	12% (3)
Hispanic or Latino	Yes	8% (2)
	No	92% (24)
Years of clinical practice in UCD	<3	8% (2)
	4‐6	25% (7)
	7‐10	16% (4)
	>10	47% (12)
	Do not know/not sure	4% (1)

### 2.4 Data analysis

Thematic content analysis was utilised to categorise data into recurrent themes. Initial data abstraction was accomplished through line‐by‐line open coding of a cross‐section of 14 interview transcripts by 3–4 coders. Open coding was utilised to generate a preliminary codebook and refined through team consensus until a final structure of codes emerged. This coding structure was applied across all transcripts. Data collection and analysis were conducted through an iterative process until analytic saturation was reached.

Selective and axial coding were applied to move beyond a typology of participant accounts to identify core concepts and explore the relationship between key themes. To facilitate this level of analysis, common qualitative analysis techniques such as charting and mapping and interpretation were employed.^38^

All interview and focus group data were managed using QSR International NVivo (v. 11) software.^39^

All procedures followed were in accordance with the ethical standards of the responsible committee on human experimentation (institutional and national) and with the Helsinki Declaration of 1975, as revised in 2000 (5). Informed consent was obtained from all patients and providers for being included in the study.

This article does not contain any studies with animal subjects performed by any of the authors.

## 3 RESULTS

### 3.1 Context of limited empirical evidence

Interviews and focus groups captured a context of insufficient evidence and ill‐defined clinical guidelines with regard to the choice between MM and LT. Parents and providers described challenges of treatment decision‐making against a backdrop of high uncertainty and detailed a decision‐making experience largely defined by this context (Appendix, Box 1, [1a‐c]). The decision‐making framework was constructed within this landscape.

### 3.2 A framework for treatment choice in UCD

**Figure 1:**
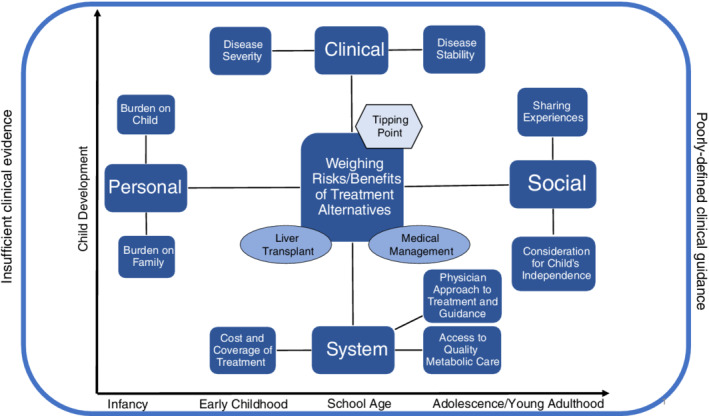
A conceptual framework describing the key factors that contribute to the decision between MM and LT among families whose children are diagnosed with UCD, within a context of insufficient clinical evidence and poorly defined clinical guidance

Key themes were positioned within a framework that illustrates how factors interrelate and collectively influence the decision between MM and LT (Figure 1).

### 3.3 Weighing the relative risks and benefits

Consideration of the relative risks and benefits of MM vs LT was a central component of the decision‐making experience and thus, positioned at the core of the framework. Parent participants described their efforts to understand and compare risks and benefits (Appendix, Box 2, [2a]) while struggling to weigh treatment alternatives in the absence of evidence‐based guidance (Appendix, Box 2, [2b‐d]).

In the absence of uniform clinical guidance, families relied on experience (their own, their providers', and their peer's') as inputs of imperfect information. Participants discussed these inputs as an inter‐related collection of complex clinical, personal, social, and system factors. These factors, found on the four cardinal points of the framework, inform each family's personal perception of the risks and benefits of MM vs LT, and ultimately, their decision to pursue or not pursue LT as a treatment for their child.

### 3.4 Tipping point

Parents who had chosen LT as a treatment for their child all reached a “tipping point” in their evaluation of the risks and benefits of LT vs MM. Ultimately, these families felt unable to continue managing their child's disorder through diet and medication, prompting (ie, “tipping”) them to pursue LT (Appendix, Box 3 [3a]). Interviews highlighted variation in how families approach this decision and the conditions under which they entertain transplant as a viable treatment alternative. If, when, how, and for what reason families reach this conclusion varied within the study cohort. Some described their “tipping point” shortly after diagnosis, some never reached a point where transplant was a true consideration, and others faced their “tipping point” after years of MM and key changes in circumstance (Appendix, Box 3, [3b‐d]). The family's tolerance for the uncertainty that accompanies MM (Appendix, Box 3, [3e]), the child's clinical status, the personal burden of disease, the social implications of the illness, and the patient's experience with the health‐care system each factored into this timeline to various degrees. Together, these represent the landscape within which families affected by UCD evaluate available treatment choices and reach or do not reach a “tipping point” in favour of LT.

### 3.5 Clinical factors

#### 3.5.1 Disease severity

The severity of the child's diagnosis was cited as a key consideration in the choice between MM and LT. For patients with neonatal‐onset UCD, who are presumed to have virtually zero enzyme function, LT was often presented as the more evident choice. In these cases, parents and providers described transplant as the child's “only option” or “best chance at long‐term survival” (Appendix, Box 4, [4a‐b]).

It is important to note, however, that despite what most participants described as a compelling clinical argument in favour of LT for the most severe patients, some families still expressed hesitation in pursuing LT and continue to evaluate the potential complications of surgery against the risks of MM (Appendix, Box 4, [4c]).

In cases of late‐onset UCD or partial enzyme function, where MM is presented as a viable treatment alternative, participants described a much more subjective evaluation of the pros and cons driven by factors outside that of the child's diagnosis (Appendix, Box 4, [4d]).

#### 3.5.2 Disease stability

Study participants differentiated between the child's disease severity, determined by their diagnosis, and the stability of their disease, reflected in the family's ability to control the child's ammonia through diet and medications. Parents often pointed to a period of frequent hyperammonemia and hospitalizations as a catalyst for transplant (Appendix, Box 5, [5a]). In some cases, disease control was never truly established (Appendix, Box 5, [5b]). Other families described a sudden loss of control over their child's ammonia (Appendix, Box 5, [5d]).

Among children who experienced fewer hospitalizations, some parents viewed LT as a last resort (Appendix, Box 5, [5e]) while others considered transplant as a preventive measure to avoid future complications. In these cases, parents did not interpret past disease stability as a predictor of future disease control, citing the unpredictable and potentially devastating nature of high ammonia as a driving force in their decision to pursue LT (Appendix, Box 5, [5f‐g]).

### 3.6 Personal factors

#### 3.6.1 Burden on family

Parent participants extensively discussed the day‐to‐day challenges of managing their child's illness and the ways the disorder has altered their family life. Parents called attention to 24/7 medical caregiving, the impact of fear and worry on the family's emotional health, and the family's altered relationship to “normal” life comforts like food and travel. For many, these daily burdens provided a compelling reason to consider LT (Appendix, Box 6, [6a‐c]). For other parents, day‐to‐day challenges, although present, were not enough to prompt them to pursue transplant. In these cases, parents often described reaching a point of mastery and comfort in their child's MM routine and cited concerns about the new and unfamiliar risks of post‐transplant life (Appendix, Box 6, [6e‐f]).

#### 3.6.2 Burden on child

Parents also discussed the burden of illness on their child's quality of life. Many participants expressed deep concern over how UCD impacts their child's intellectual and social development. Parent's often labelled their children's demeanour in immeasurable terms such as “foggy,” “unfocused,” “disorganised,” or “cloudy,” and worried their child was suffering due to heightened levels of ammonia. Those who considered LT often believed the surgery could offer their child an opportunity at a “normal” and better‐quality life (Appendix, Box 7, [7a‐c]). Among those who did not pursue LT, some described a much more optimistic picture of their child's current intellectual and social growth, including participation in school and sports (Appendix, Box 7, [7e‐f]).

### 3.7 Social factors

#### 3.7.1 Peer‐to‐peer interaction

Parents reflected on conversations with other families affected by UCD and the role their peers played in shaping their own treatment choices. Most parents interacted, to varying degrees, with other families affected by UCD. While many pursued connections to other parents for the specific purpose of informing treatment choice, others connected organically through the UCD community. Regardless of how connections were made, many parents described being influenced by others' experiences with MM and LT. Parents were motivated to transplant based on the positive surgical and post‐surgical experiences of some families (Appendix, Box 8, [8a‐c]) and the negative outcomes shared by others who had delayed or foregone transplant (Appendix, Box 8, [8d]). Other parents were deterred from LT by stories of surgical complication (Appendix, Box 8, [8e‐f]) and/or encouraged to continue MM by others who had done so with success (Appendix, Box 8, [8 g]).

#### 3.7.2 Consideration for child's independence

Parent participants often discussed their child's independence as a point of continuous concern. Parents considered shorter‐term steps towards independence like participating in school programs as well as longer‐term goals like living outside the home and attending college. Most parents did not trust others to manage their child's strict dietary regimen. Others worried that rising ammonia levels would impede their child's ability to recognise a crisis and take appropriate action. For many parents, transplant represented the only viable way to remove the threat of hyperammonaemia and ultimately, afford their child independence (Appendix, Box 9, [9a‐b]).

Other parent participants offered a different perspective on living independently with UCD. These parents described incremental efforts aimed at teaching their child to manage their medical needs. They shared a belief that their children could live safe and independent lives with UCD and thus, were not driven to pursue LT by these specific concerns (Appendix, Box 9, [9c‐d]).

### 3.8 System factors

#### 3.8.1 Access to quality metabolic care

Parents considered their level of access to quality metabolic care when weighing treatment options. Parents who lacked confidence in their local metabolic team often pursued transplant to address what they perceived as inadequacies in their child's long‐term and emergency medical care (Appendix, Box 10, [10a‐b]). This issue was further framed by the family's geographic residence and their relative proximity to specialised UCD services. Families who lived farther from a hospital centre with expertise in UCD worried about timely and appropriate rescue care during hyperammonaemia. In some cases, this fear was a key driver in the parents' decision to pursue LT (Appendix, Box 10, [10c‐d]).

Parents who conveyed satisfaction with the local long‐term and emergency metabolic care options available to them expressed greater confidence in their physician's ability to help control their child's ammonia and in the hospital's capacity to address medical crises. These parents were often less motivated to explore alternatives to MM (Appendix, Box 10, [10e]).

#### 3.8.2 Metabolic physician approach to treatment and guidance

Physician approach to treatment guidance held influence on parent treatment choice. Many parents described the relationship with their metabolic doctor as critical to their child's care and welfare. Thus, guidance from the metabolic doctor in favour of or against LT was highly valued by many parents (Appendix, Box 11, [11a‐b]). In the absence of information specifying the conditions for one treatment path over another, physician approach to treatment varied substantially from person to person. Our data suggest that these differences are driven, in part, by the physician's previous experiences with LT, the location of their training and that institution's general position on LT for UCD, and the outcomes they observed among their MM patient pool (Appendix, Box 11, [11c]).

Some families credited their physician's staunch opposition as a major deterrent to transplant, even in circumstances they now feel may have warranted it (Appendix, Box 11, [11d‐e]). Other parents were encouraged by their physician to explore transplant as an alternative treatment choice, leading some to pursue it (Appendix, Box 11, [11f]). Still, other providers described a position neither for nor against transplant. Some families valued this impartial approach, feeling empowered to explore both treatment options with the support of their metabolic doctor (Appendix, Box 11, [11 g]). Others described feeling paralysed by their provider's ambivalence and wished that their doctor had done more to assist them in weighing treatment alternatives (Appendix, Box 11, [11 h‐j]).

#### 3.8.3 Cost and coverage of treatment

Parents cited costs of care and the burden of navigating insurance coverage as a major challenge. Qualitative data highlighted clear differences in the cost and coverage of MM vs LT. Parents described transplant as a fully covered procedure with little out‐of‐pocket cost for surgery, hospital stay, short‐, and long‐term post‐transplant medications, and follow ups (Appendix, Box 12, [12a‐b]). In contrast, parents described on‐going struggles with the cost and coverage of their child's pharmaceutical and nutritional needs under MM. Parents cited time‐consuming disputes over coverage for medications, metabolic formulas and medical foods, and indirect financial costs related to travel for medical care and reduced time at work (Appendix, Box 12, [12c‐d]). Despite these differences, parents did not point directly to finances as a driving force behind their treatment choices. However, for many participants, these financial implications contribute to the overall burden of disease and the context within which treatment decisions were made.

### 3.9 Phases of childhood and developmental milestones

Qualitative data demonstrated that changes during key developmental milestones precipitate new and/or aggravate existing challenges associated with the MM of UCD, acting as a catalyst for parents to consider for the first time or reconsider LT as a viable treatment choice. For example, as a child moves from infancy to early childhood, parents contend with new feeding challenges, including a transition to solid foods (Appendix, Box 13, [13a‐b]). They also face adherence issues once their child is able to refuse medications, formulas or other forms of nutrition (Appendix, Box 3, [3c]). Parents have an increasingly difficult time protecting their child from viral exposures as they transition to school age (Appendix, Box 13, [13d]) and face new challenges managing UCD in a school setting, including concerns about forging peer relationships and participating in “normal” childhood activities (Appendix, Box 13, [13e]). During adolescence and early adulthood, parents cited new adherence issues (Appendix, Box 13, [13f]), questions about their child's long‐term independence, and rising concerns about their child's ability to manage their own medical needs (Appendix, Box 13, [13 g‐h]). Data suggests that the clinical, personal, social, and system factors that influence treatment choice, manifest differently across these key phases of childhood. Thus, we include these major developmental transitions and milestones on the “x‐” and “y‐axes” of the framework to indicate that they may change priorities and re‐frame the parent's perception of risks and benefits.

Table 3 provides a summary of the key concepts outlined above with select exemplary quotes to reflect the perspectives of parent and provider participants. Additional quotes to support our analysis can be found in the Appendix (Box 1‐13).

**Table 3:** Summary of key concepts related to the treatment decision‐making experience of families affected by UCD and exemplary patient and provider quotes

Concept/domain		Exemplary quote
Context of limited empirical evidence		Provider: “We need to get more data to know what we are doing…I think it's lack of data and knowing if we are doing the right thing for this child or if we are actually harming them more than we are helping.”
Weighing risks and benefits of treatment alternatives		Parent: “It's the same thing as a risk benefit. You're making a pro and con list, and it's an unknown number of hyperammonemic episodes vs unknown complications from liver transplant.”
Clinical	Disease Severity	Provider: “I think in the severe neonatal onsets; I think that's less of a question at this point. That's really the only way to save them…In the later onsets, where it's a little bit less clear‐cut, I think—we have extensive conversations.”
	Disease Stability	Parent: “Initially, we were not for transplant…I just saw all the complications and the constant taking of medication…We thought, oh, we can keep him managed, but basically, it started getting to the point where [he] was beginning to have to be hospitalised every couple of months for illness.”
Personal	Burden on Family	Parent: “It really impedes your life, your family, and I would not want that for any new family. If we could protect them and they do not ever have to go through it, and if transplant is safe…That's the best option…the lack of sleep and constant worry, completely sleep deprived because you check to make sure they are fine all night long. The stress of what if something happens, that takes many years off your life.”
	Burden on Child	Parent: “He learned how to count money, and that was a huge thing because he worked and worked at it. Then he had a high ammonia level…He remembered that he knew how to count money, but he could not count it anymore. We thought, oh, that quality of life's horrible…He had to work so hard to learn it more than just a normal kid, and then to lose that functionality was devastating for him. That played into [the decision to transplant] too.”
Social	Peer to peer interaction	Parent: “A good friend of mine lived nearby in. Their daughter was transplanted. She died…That left a bad taste in my mouth…for a long time we did not even really give [transplant] much thought.”
	Consideration for child's independence	Parent: “For her independence, a transplant is necessary…when her ammonia level starts to rise, she cannot make decisions on how to help herself…. Living on her own and going away to college was not going to be an option.”
System	Access to quality metabolic care	Parent: “We're here…with very limited access to a decent metabolic geneticist …It seems that there are only a handful of specialists throughout the country, and if you are not in that location, you are really subject to pretty subpar care…I think the question had to do with local—not having local access to good physicians. We never felt like they had our backs here…so that was a huge stress for me knowing that we were basically on our own.”
	Physician approach to treatment and guidance	Provider: “People have very different approaches at different institutions…. I think it has a lot to do with, especially if you only have a few cases and then you have even fewer cases who decide to go through transplant, what happens to them afterwards. If you see a bad outcome or two that can totally change your impression for the next 20 years vs if you see some who do really, really great, then that may also change your referral pattern.”
	Cost and coverage of treatment	Parent: *“*In the very beginning I had to do a lot of navigating with [my child's] medication…They did not want to cover it…I spent many hours on the phone…The actual cost and coverage with [his] transplant, we have not had to worry about that at all. That was covered.”
Phases of childhood and developmental milestones		Provider: “Especially with the older children…who's going to manage the child who does not have a liver transplant after you are dead and gone or if you become incapacitated?…With the younger patients, I usually do not take that approach, but as the patients get into their teenage years, it's a question of, well, who is going to manage this?…That's something that really is important to think about.”
Tipping point		Provider: “Finally, push came to shove where it was the kids were coming in too frequently, or their ammonias were being too problematic, difficult to treat. Then we finally made the decision when that balance or the scale seemed to tip.”

## 4 DISCUSSION

This paper presents an original framework that reflects various inputs to a highly complex and dynamic personal evaluation of the risks and benefits of treatment alternatives among families affected by UCD. This study is novel in that it examines an understudied area of rare disease and provides a model for research around treatment decision making in rare disorders that may be applied to other conditions.

Although there have been no commensurate publications examining treatment decision making among families affected by UCD, several of the factors identified through this work, including the influence of peer‐to‐peer interactions, provider recommendations, and developmental milestones, align with and augment previous research on decision‐making in paediatric transplant.^25^, ^26^, ^35^, ^37^ A description of this previous research and its alignment with concepts identified through this study can be found in the appendix (Table A3).

This study also contributes new evidence supporting the role of other factors, not previously described, in driving treatment decisions in UCD. This study distinguishes between the function of disease severity and disease stability in mediating treatment choice, expounds on the implications of disease for the family and the child and its role in treatment decision‐making, and explores issues of health care quality, cost, and access as they relate to the choice between MM and LT.

Since no previously published studies have examined these factors in terms of their impact on treatment choice in UCD, providers have relied on anecdotal experience in guiding their understanding of how these issues bear on treatment choice. The role of these factors, particularly those unrelated to clinical markers of disease severity and stability, such as coverage, cost and access to medical vs surgical treatment generally function outside the purview of the clinician and so are likely underweighted by many medical providers. Thus, the results from this analysis hold practice implications for members of the patient care team. This framework equips providers with an evidence‐based account of the patient experience so that they may better address the concerns, needs, and expectations of patients and families during treatment counselling.

Patient‐centred outcome measures (PCOMs) are characterised by our ability to evaluate outcomes that reflect patient needs and priorities.^40^ Historically, the effectiveness of most rare disease interventions has been determined by the evaluation of surrogate clinical outcomes that may not reflect the benefits that patients most value.^41^ The treatment decision‐making framework constructed through this analysis begins to meet the objectives of PCOM development in rare disease by defining what families value most in terms of alleviation.^42^, ^43^ Future studies may build on this research to develop quality of life metrics that better capture the unique personal, clinical, social and system burdens of UCD and its related therapies. If developed, these outcome metrics could offer more meaningful estimations of patient benefit and thereby reduce uncertainty over the effectiveness of treatments for UCD.^40^

## CONFLICT OF INTEREST

The authors declare no potential conflict of interest.

## AUTHOR CONTRIBUTIONS

M. T. G. led the development of the study design and protocol, collection of interview and focus group data, qualitative analysis, and manuscript preparations for this research. A. R. M. participated in all aspects of this research including providing guidance on study design and protocol, groundwork for data collection and analysis and substantial manuscript revisions. K. Z. G. provided substantial research support in the analysis and interpretation of qualitative data and participated in manuscript revisions. D.M.S. provided substantial guidance in the interpretation of qualitative data and significant contributions during manuscript revisions. C.L.M. provided substantial guidance in the conception of the study and protocol design, supported recruitment of study subjects, and provided manuscript revisions. J. B. supported the development of the study protocol, co‐led recruitment of study subjects, and provided manuscript revisions. N.A.M. was PI for this study, providing guidance on all aspects of study design and protocol and substantial manuscript revisions. All authors will provide approval for the final version of this manuscript prior to publication and agrees to be accountable for the accuracy and integrity of this work.

## APPENDIX 1

**Table A1** A description of currently available treatment guidance and evidence to support treatment decision‐making in urea cycle disorders (UCD)

**Table:**

Intervention type	Content of clinical guidelines	Evidence to support effectiveness
Medical therapeutic interventions for UCD	Current treatment guidelines focus on clinical diagnosis of UCD, management of acute hyperammonaemia, long‐term management through diet, medications, and amino acid supplementation, monitoring of patients, and cognitive outcomes and psychosocial issues. These guidelines were developed through professional consensus using the GRADE methodology for scoring evidence levels. However, the evidence available to support UCD guidelines are predominantly non‐analytical (eg, case series analysis, case reports).^15^	A few key large retrospective and prospective cohort analyses in UCD have aimed to determine the effect of medical therapeutic interventions for UCD on various outcomes including survival and cognitive function. 3, 6, 16, 17, 46 Results of these clinical studies must be compared with caution, since disease severity within study cohorts often differ and treatment combinations vary widely across providers and institutions.
Liver transplant as treatment for UCD	Current guidelines discuss liver transplantation as the only available curative treatment for UCD and suggest that transplant be considered for patients with severe UCDs who are not responding to medical therapy, experience recurrent metabolic decompensations requiring hospitalization, report poor quality of life, and are without severe neurological damage. 15	Although liver transplantation is performed in increasing frequency to treat UCD and survival rates seem to have improved over time with surgical advances, 18 there is not a great deal of data on neurocognitive function post‐transplant among patients with UCD or a robust evidence‐base comparing the relative value of liver transplant over conventional medical management techniques. The psychological, social, and health problems resulting from successful childhood transplants are also only beginning to be studied and recognized. The life expectancy among young children who have been transplanted is not well documented and little information is available on long‐term complications and quality of life in this group. 19, 20

**Table A2** Limitations of the study sample

**Table:**

Characteristics of sample	Limitation
Most caretaker recruitment was conducted via NUCDF, a non‐profit advocacy organisation for UCD patients and a resource of information and education for families affected by UCD.	Study sample may not capture the perspective of individuals who have not engaged on some level with this organisation.
Parent participant sample is skewed towards a predominantly white, educated, and affluent demographic.	The experience of these individuals may differ systematically from the experience of those who were interviewed. In the future, recruitment source and strategy should be diversified to capture the perspective of traditionally underserved and vulnerable populations affected by UCD, which are currently under‐represented in our sample.
Given the sensitive nature of their experience, parents whose child had passed away from complications related to UCD or liver transplant were not interviewed.	Study sample may not capture the perspective of individuals who lost a child in response to either treatment choice. Thus, may omit key elements of their experience.

**Box 1: Context of limited empirical evidence**

*[1a] Parent participant*: “The most difficult part, I guess, is the unknown…. I think the most difficult piece of it was just the lack of information in one location. We had to go through so many avenues to get the information that I needed to feel better about the decision that we were making. I needed all the information, and we had to go everywhere…. That's the hardest part. It's so hard to make the decision when you do not have all this data.”
*[1b] Provider participant*: “I think just the uncertainty of what is going to give their child the better long‐term outcome is really hard…The fact that I cannot totally resolve that uncertainty.”
*[1c] Provider participant*: “Lack of us knowing what we are doing [is most challenging] …we need to get more data to know what we are doing. Are we really helping them or are we doing more harm: Do we need further stringent criteria to decide on this or is what we are doing okay: I think those are the questions that we need to answer…I think it's lack of data and knowing if we are doing the right thing for this child or if we are actually harming them more than we are helping.”

**Box 2: Weighing the relative risks and benefits of available treatment options**

*[2a] Parent participant*: “We wanted to be in the driver's seat…for his best interest and his life…it's the same thing as a risk benefit. You're making a pro and con list, and it's an unknown number of hyperammonemic episodes vs unknown complications from liver transplant. I think it's a hard decision, especially in those that might not be extremely sick right now.”
*[2b] Provider participant*: “Most challenging for the families—I think…they are probably, in many cases, wrestling that risk/benefit ratio. They're saying, ‘How much chance do I want to take with my baby:’ When we tell them how sick their baby can get from hyperammonemia, and then they hear from the surgeon how tricky the surgery could be… I see that as being a tough balance to accomplish.”
*[2c] Provider participant*: “I think the risk‐benefit is much easier for families to see in kids that get transplanted for other things…where the kid is literally getting sick before your eyes in the hospital…we can tell you rather definitively that the only way your child is going to survive is to get a liver transplant as soon as possible. I think those decisions are just easier to make. The risk‐benefit is much easier to assess because you can see how sick the kid is and how much medical support they are requiring… it can be much harder [in UCD] because the child looks okay and you are going along life as you have been doing and they have limitations but you get used to it. It's a really different decision, I think.”
*[2d] Parent participant*: “It just seems like— because there's no standard practice in these cases, it's extremely difficult. It feels like we are on the frontier of this thing, and it's not clear what the right decision is…we feel like we are making a decision, and we do not really have all of the information…Maybe that's just because that information does not exist.”

**Box 3: Tipping point**

*[3a] Provider participant*: “Finally, push came to shove where it was the kids were coming in too frequently, or their ammonias were being too problematic, difficult to treat. Then we finally made the decision when that balance or the scale seemed to tip… Every child is different, and every child comes in with a different set of issues. Whether it's the diet, whether it's the medicine, whether it's just the lack of activity or just feeling out of it all the time, whether it's hyperammonemic crises, bringing them in frequently, these are all issues that come into the play of whether or not you go ahead and you finally make a transplant decision.”
*[3b] Provider participant*: “Families take that information in different ways. I'll have kids that I think have a little bit milder disorder, where we have talked about transplants because I usually do in the urea cycle defects. I at least let them know what the spectrum of options are…The family, well, when can we do the transplant: I say, your kids aren't that sick. Then we have other ones where the kid's coming in once a month, and they have had many [hyperammonemia episodes] …and the families say, oh, I do not know if I want a transplant.”
*[3c] Provider participant*: “For some parents it's right away. They want the transplant right away. They want this condition gone. This condition scares them way more than a transplant. There are some parents who, within a year, would say, ‘I want a transplant.’ … Some families just reach it right away and others have to sit for a while with it.”
*[3d] Parent participant*: “It was his second crisis then at 10 months, I guess,…that tipped the scales…. After the second crisis, there was—we did not feel like it was a—it was a decision that was definitely transplant.”
*[3e] Parent participant: *“Honestly, as parents we were not handling it well. We were handling it but to think of living years like that, with that kind of fear, was overwhelming…. I think for us it was the fear of her just having a completely severe decompensation to the point where she'd have a really severe brain injury and that her life could completely change in an instant… I think it's one thing that if your baby's born a certain way and that's what it is and you deal with that situation. When you are just constantly facing it but knowing that you are not there but that everything could change for them at any time, to me it's just a really different way to try to exist with just the fear of it.”

**Box 4: Disease severity**

*[4a] Parent participant*: “He had almost a compete deletion in his chromosome. Anything could just set it off…They were like ‘It can go off anytime. It can cause another hyperammonemic episode… He could possibly die, or go into a coma, or have seizures…This is his best chance of survival.’ That's what we did….it was really the only long‐term option for him.”
*[4b] Parent participant*: “[He] had 0% of the OTC enzyme. Honestly, we really were not given a choice. It was sort of a no‐brainer like, okay, you live in fear for the rest of your life that when he gets sick his ammonia will go up and cause further brain injury. That's what we chose really with the guidance of the metabolic team.”
*[4c] Parent participant*: “I do not really know. I feel like she's probably always going to be a little bit unstable. We seem to be in and out of the hospital every three months, about. I do not know. She has zero enzyme function, so it's pretty black and white in her case…. I feel like things probably aren't going to change very much, but who knows:… Do we do it now and protect her neurological development, but then risk her life, or do we continue with conservative management in the hope that maybe an alternative treatment's going to develop:”
*[4d] Provider participant*: “I think in the severe neonatal onsets; I think that's less of a question at this point. That's really the only way to save them….I think that in that case, there's…less question about whether it's the right thing to do…In the later onsets, where it's a little bit less clear‐cut, I think—we have extensive conversations.”

**Box 5: Disease stability**

*[5a] Parent participant*: “He was in the hospital every week or so because his ammonia would shoot up, and we really had no control over it. No matter what diet, how we adjusted his diet or his medications, his ammonia would always just go up. It was necessary for him to get a transplant because we could not really control his ammonia.”
*[5b] Parent participant*: “In her case, we just could not keep her stable. She was pretty stable for two months before she was hospitalised again, but she had a handful of decompensations within the first few months. We were already feeling pretty desperate in talking about transplant within the first three months of her life”.
*[5d] Parent participant*: “Initially, we were not for transplant. I, of course, did all the Google stuff, which I know you probably should not do, but I just saw all the complications and the constant taking of medication. It just did not seem like something that we wanted to do. We thought, oh, we can keep him managed, but basically, it started getting to the point where [he] was beginning to have to be hospitalised every couple of months for illness.”
*[5e] Parent participant*: “From everything that I've heard, it should be for us more of a last resort scenario. My daughter's condition, for the most part, has been pretty well‐controlled. She's had some, a few, maybe three or four high ammonia episodes in the last ten years that required hospitalisation, and those were mostly well‐controlled. In my mind, if that is still feasible for us, why would we take on the risks that are associated with the liver transplant:”
*[5f] Parent participant*: “Tons of reasons as to why we felt transplant was a better option, even though he was quite a stable kid so far, but everybody told me that might not be the case forever. That can change literally overnight, so that's why we decided to go for the transplant.”
*[5g] Parent participant: *“She is stable. She's met all her developmental milestones. She is thriving. You look at her, and she is very typical…So when she went into the [transplant] doctor's office, they are looking at us like, okay, she's fine right now…but what about when she has her next one and…what is that going to look like: That terrifies us.”

**Box 6: Burden on familial unit**

*[6a] Parent participant*: “I'm like, oh my God, she never would've been able to do this…It would've impacted our lives, as well…just your daily life and meal planning. ‘Those little things that I sit and think about, when I get overwhelmed with anything going on in her life now, I'm like, what would it have been without [transplant]:’ and then I'm like, okay, I'll take it.”
*[6b] Parent participant*: “Before it was every night I never left things undone because it was always, oh, is tonight a night we are going to have to race off to the hospital:…Like I said, [transplant] changed our lives in a lot of ways.”
*[6c] Parent participant*: “It really impedes your life, your family, and I would not want that for any new family. If we could protect them and they do not ever have to go through it, and if transplant is safe, with it being as safe as it is, then that's always an option. That's the best option…the lack of sleep and constant worry, completely sleep deprived because you check to make sure they are fine all night long. The stress of what if something happens, that takes many years off your life.”
*[6e] Parent participant*: “That has definitely been a challenge, just to have a healthy amount of and realistic amount of worry, not to let yourself fall into that pattern of panicking…this condition is not for the faint of heart. Managing it, I'm not going to say it's easy by any means, but it is doable…Despite all of our ups and downs and things we have to go through when we think she's sick, I'd still rather deal with that than have to deal with X% dying and all the issues that come with having a major organ transplant… I just do not see a reason. The path unknown is really frightening to me, and there's no reason for us to go down that path unless we need to.”
*[6f] Parent participant: *“Right now, he is living a 100% functional, wonderful life. Getting a liver transplant would just be replacing his urea cycle disorder with another condition. He would be on…immunosuppressant drugs for his whole life. I feel the way I manage his urea cycle disorder; I would have time if something were to happen. If he were to get the flu, or some horrendous illness that would spiral his urea cycle disorder into a horrible ammonia attack, we'd have time to deal with that….If he gets the flu, I can usually manage it from home…I feel like if it was a liver rejection, and say there wasn't another liver available, I would not have all these options. I would not have the options to save him the way I could now with a urea cycle disorder. That's why I've chosen not to transplant him.”

**Box 7: Burden on child**

*[7a] Parent participant*: “I remember she would wake up every morning, and she'd be just moaning…I do not know if it was pain or if it was…just foggy ammonia brain…. She was just never normal…. It was just very ‐ I do not know. It was very sad…It was day‐to‐day. It was hell. She would be stuck to a pump for an hour, and then we were still at that time trying to feed her orally. We'd sit her down at the table, and she'd sit for hours just trying to get a little bit of food in, and then by that time, it was time for another feeding. I mean, she had no life. She could not play. I mean, she was miserable…her thoughts were disorganised and cloudy. She wasn't a normal kid. It was heartbreaking.”
*[7b] Parent participant*: “That was one of the things in school. He learned how to count money, and that was a huge thing because he worked and worked at it. Then he had a high ammonia level…He remembered that he knew how to count money, but he could not count it anymore. We thought, oh, that quality of life's horrible. That was another one of the things that—especially he had to work so hard to learn it more than just a normal kid, and then to lose that functionality was devastating for him. That played into [the decision to transplant] too.”
*[7c] Parent participant*: “We did not let her do a whole lot outside of our house, in terms of being normal, regular kids that can go play, and go swim, and go to school. We were too worried constantly about what she was going to get into, or was she going to burn too many calories, or was she going to wear herself out. We just kept her in a bubble. It was very stressful.”
*[7d] Parent participant: *“He's very active. He's very involved in sports…he plays football. He plays basketball. He plays lots of sports. [The doctor] adjusted his metabolic formula. She adjusted some things…He, obviously, is gaining weight. He's growing. His height, he's getting taller. He's doing so much better.”
*[7e] Parent participant: *“Of course, there's worry, but she does not live in a bubble and she never will. I want her to be out and about and to be part of normal life. It's just she's going to have to take more precautions than other people.”

**Box 8: Peer‐to‐peer interaction**

*[8a] Parent participant*: “I probably wrote back and forth with probably about six, or seven, or eight different moms for a couple months. Just hearing their experiences, asking if they feel that dealing with transplant life is easier than a UCD life, and all of them told me that they absolutely thought the transplant parent life was better than UCD life. A lot of their opinions really helped make my decision.”
*[8b] Parent participant: *“Recently, a girl that is 14 that's had a really hard time for the past 5 years, she was transplanted. That's someone that is close to [my daughter's] age, someone that [my daughter] has known… Seeing that and hearing how well she's doing, I think that's going to make her process a little easier.”
*[8c] Parent participant: *“There is a girl…We met her at a urea cycle disorder conference…Wonderful young girl and she got a liver transplant, and she is doing wonderful…It's neat because [my daughter] is like you are just like me. You're just like me.”
*[8d] Parent participant: *“I had talked to one parent who had lost her child with ASA when she was 18 years old, and other parents whose kids' livers were doing really poorly in their teens, and I thought, I should just preempt this now. I'm not going to do this struggle of trying to get him to have nutrition, watch his brain deteriorate when it's not preventing the hyperammonemias, and just to get him to a teenager where he's going to die anyway. That made me request the transplant.”
*[8e] Parent participant*: “A good friend of mine lived nearby in. Their daughter was transplanted. She died. It was a bad ‐ things just went poorly the whole way through… I do not think she ever even came home from the hospital after being transplanted. That left a bad taste in my mouth, and between that and the fact that our doctor was very adamantly opposed to doing a transplant, for a long time we did not even really give it much thought.”
*[8f] Parent participant*: “We've lost touch with them because now they are on a different track; they are being followed…post‐liver transplant. From what I understand, it has not been an easy road for them at all. She's been stable…but it has not been easy at all. It, in my opinion, reaffirms why we never went down that path. What I've heard from other people going to various conferences is that it's trading one set of issues for another set of issues. If we do not have to find out what those other set of issues are, why would we: That's our thought process.”
*[8g] Parent participant*: “We felt like we were all alone, and then all of a sudden, these people just pop up, and here they are. They have this son who is…I think he was five at that point. It was just incredible…This mom was like, ‘Look, this could be [your son]. This could be [your son] in five years.’ It was a completely different outlook on what we were thinking…. They're handing our infant to us, and we are thinking that he's going to die. Then all of a sudden, we have this other family that says, ‘No. Look. Look. We have this son. It has not been easy but look at him. He is beautiful and healthy.’”

**Box 9: Considerations for child's independence**

*[9a] Parent participant*: “At one of the meetings at the Urea Cycle Foundation…they talked about…He was living on his own, and he died because his parents could not get hold of him. When they found him, he had had a high ammonia episode, and nobody was there…That made it an easy decision. We'd never let him live alone…When he'd get sick spending the night at his cousins, we were like heck, we do not want to let him spend the night anywhere. We do not want to take the chance…What kind of a life can he have if someone has to be watching him 24‐hours a day:…Some of those episodes came on so fast that even if he was a normal adult, he might not have been even able to call 911 in time to manage it for himself.”
*[9b] Parent participant*: “Because [she] has done so well in school and has done so well socially, going to college…that is a very good possibility, where we never really thought that was a possibility. For her independence, a transplant is necessary…It is something that with OTC and when her ammonia level starts to rise, she cannot make decisions on how to help herself. There always has to be someone there to help her identify and help her make changes to her diet and to her medication before it gets too bad. Living on her own and going away to college was not going to be an option.”
*[9c] Parent participant: *“It's a constant struggle as I'm trying to prepare her to be a young adult, understanding that in order for her to live her life, she has to be her own caretaker. That's another huge hurdle of what we are going through right now, saying, ‘Okay, this was your responsibility. We're not going to be here all the time to monitor what you eat, whether you are taking your medicines,‘ and right now that's the biggest challenge we are having as a family…Like I tell her, if she wants to go off to college and wants to have a job, I'm not going to be there to ask her what she's eaten or whether she's done her medicine; it's all going to be on her. It's definitely a process. She's not fully aware yet, but there are signs that she's finally getting it.”
*[9d] Parent participant*: “Even right now, she's 9 and in the third grade. We're trying to transition into getting her to read food labels. Getting her to understand what everything—right now I mix up her medications, and I measure everything. We already talked about, okay, how old does she need to be before she starts getting old enough to do that: Yeah, that's our goal. We understand some day she's going to leave the nest. She's going to need to know how to do all of this. At one point do we start teaching her?”

**Box 10: Access to quality metabolic care**

*[10a] Parent participant: *“Yeah, the quality of care. Part of my decision [to transplant] too was UCD being so rare. What I was told is I cannot be on vacation and be sure to find somebody that's even going to know what I'm talking about. Even our local hospital did not even ‐ they had no idea what a UCD was. They had no idea how to care for him. I just felt it was really dangerous for him to live with that disorder in the future, for his life…. I did not feel like I was getting much help with the nutrition aspect, and I was worried about that for the future because it's such a huge part of keeping him healthy… Then a couple of times…he had an emergency. He had been vomiting all day, and we took him to the ER. Of course, the ER was terrible. They do not know anything about urea cycles.”
*[10b] Parent participant*: “All these little things were adding up for us. Okay, he did not have a crisis for eight months, but they are not able to quickly handle it. This is a hospital that actually knows about it. I had talked to plenty of parents whose child had brain damage because they were in a place…where the hospital did not know about it. Ours knew, and they still took days to get this going and get him stabilised….we just looked at each other and said, if we were not here watching like hawks all the time, would he survive?…When we went in there, my faith was that these people were going to save his life, and suddenly, I could not count on them to do it right. You know?… The head of transplant came in, and said again, ‘I think you need to talk to us again.’ We said, yes, we wanted to get him—we changed our minds. We want a transplant.”
*[10c] Parent participant: *“We're here…with very limited access to a decent metabolic geneticist. I've been told there just aren't that many, I guess. There certainly aren't any in this state, and so it's just access to good care, basically…It seems that there are only a handful of specialists throughout the country, and if you are not in that location, you are really subject to pretty subpar care…. Not being able to rely on your healthcare provider. I mean, I think the question had to do with local—not having local access to good physicians. We never felt like they had our backs here. We could never rely on them. We would put a call in and not—we would not get a call back for a week. When you are trying to manage day‐to‐day, it's like, oh, we got these labs back. What do we do now: You cannot wait a week. I mean, it was horrible, so that was a huge stress for me knowing that we were basically on our own.”
*[10d] Provider participant*: “If you have a medically unstable child who lives a long way from a facility with any special expertise with urea cycle disorders, he could get into trouble quite easily and not have medication at hand that they need. That obviously does change the balance of how you would recommend liver transplant… I have a [adolescent] patient who ‐ she was really well her whole life, but then…had one or two hyperammonemic episodes, not even that severe, quite moderate. Because of her geographic location, I recommended transplant because her ammonia control baseline wasn't that good. I was worried about compliance, and I was worried about her geographic location, so we listed her, and she was transplanted last year.”
*[10e] Parent participant: *“I'm confident that if she does get sick that we are in the right place. I trust the team here very much… Here, anytime there's been a question of high ammonia, they are like come in. Let us do labs. We wait in the hospital until we know what the number is…The reason [liver transplant is] not forefront in my mind is because we are with [this] department, and we are with [this] doctor. I'm very confident in our team on our day‐to‐day management and how we do everything…If it's decided that we cannot stay here… I pretty much know transplant would happen in one of two facilities, and then we'd go from there.”

**Box 11: Metabolic physician approach to treatment and guidance**

*[11a] Parent participant*: “I had such an incredible team that I really did not have to navigate it alone. They were really there for me…The doctors have been incredible…I never really had to navigate those things alone medically.”
*[11b] Provider participant*: “I think [the family's] view of transplant is impacted significantly by the impression they are getting from both the metabolic genetic team, who usually know them really well and is really involved with them, and the transplant team that they see. From what I've heard from other doctors and from families, they may get a very different impression of what transplant involves and what the long‐term outcomes are depending on who they talk to.”
*[11c] Provider participant*: “It seems like the discussions I've had with geneticists is that people have very different approaches at different institutions…. Some people have very clear ideas about who they refer for transplant, and some people do not. I do not think we know which one is the right approach…I was definitely surprised about that when I started talking more to metabolic geneticists at other centers or that had recently come from another center to ours…. I think it has a lot to do with, especially if you only have a few cases and then you have even fewer cases who decide to go through transplant, what happens to them afterwards. If you see a bad outcome or two that can totally change your impression for the next 20 years vs if you see some who do really, really great, then that may also change your referral pattern.”
*[11d] Parent participant*: “He was very, very anti transplant, very anti transplant, just really had nothing positive to say about it, and he remained that way. I will say that's part of the reason [my son] was so much older; why we did not give it more consideration…There were a few periods where he was really sick for a really long time and almost died a couple times, and still, this doctor held fast. He did not think transplant was the way to go…When I'd bring it up to the doctor, he still was not in favour of it, so we just did not really push…. I still was not positive it was the right ‐ at that point, I still was a little unsure. I mean, having a doctor for 20 years that is the top of the field saying do not do it. Do not do it. It's like, oh, man, are we asking for trouble doing this:”
*[11e] Parent participant: *“I would say, for our family, one of the biggest challenges was that we did not have support from our genetic team… They were not encouraging a transplant. [The hospital] believes that you are much better to manage the disorder. Because we trusted these people because they saved her life and because they kept her alive, we had our full trust in them with how to handle her. Then when we decided to look into transplant, and it wasn't even something that we could bounce ideas off of them; it was something we just did not talk about. I would say that was, for us, one of the bigger challenges.”
*[11f] Parent participant*: “We mostly spoke with the geneticist…and if you are familiar with him at all, he is of the mindset that if you are born with OTC, basically, you need a transplant. With that mindset, it just ‐ it was like this is happening. This is the best solution. Oh, yeah. By the way, there are risks, and here is what they are. It wasn't even like a risk benefit analysis so much. It was just be prepared. These are the things that could happen, full well knowing that this is going to be a far better choice for your daughter at this stage. That was already the foregone conclusion, I think, when we arrived.”
*[11g] Parent participant*: “We did pursue transplant. [Our doctor] has just been open to what we as parents feel comfortable doing. He thought it was important for us to explore and see what would be beneficial for [our son].”
*[11h] Parent participant*: “It just seems like ‐ because there's no standard practice in these cases, it's extremely difficult. It feels like we are on the frontier of this thing, and it's not clear what the right decision is, and nobody really wants to say one way or the other.”
*[11i] Parent participant*: “I mean, if I could change it, I really wanted to have more guidance from [my son's] metabolic doctor, but he never really gave much of an opinion.”
*[11j] Provider participant*: “What we often tell the family is that there's not a right answer here. It's not wrong to choose one or the other. It's what's right for them as a family together. That, I think, is difficult for families because in something so major, I think what they really want to hear often is, ‘This is what you should do. This is what you need to do for your child.’ Making that decision and putting it on a family is really difficult. I always feel that when we have these discussions that I wish I could just tell them what to do. I think that would take a lot of the burden off the family.”

**Box 12: Cost and coverage of UCD**

*[12a] Parent participant*: “In the very beginning I had to do a lot of navigating with [my child's] medication, the sodium phenylbutyrate…They did not want to cover it…I spent many hours on the phone for about a month after [he] was born, having to get him what he needed…The actual cost and coverage with [his] transplant, we have not had to worry about that at all. That was covered. We never got any issues and trouble for it.”
*[12b] Parent participant*: “The transplant coverage has been beautiful…After surgery, we accidentally left [medication] in our refrigerator, and we went [on vacation]…Even though we were out of state, the insurance company was more than willing to pay for a small amount of medicine to be made right there…at 10 o'clock at night. Otherwise, we were looking at turning around and coming right home. That's what we would've had to do, and we were prepared to do that…The insurance was like, no, no, we'll pay for five days' worth.”
*[12c] Parent participant*: “It's too hard…insurance, and the cost, and the struggle between insurance, and where you work, and getting covered, and it's not just one medication, it's multiple, it's a full‐time job just to get medication. The amount of hours that I have spent just to get medication sent to our front door, we have driven all over the place and had things flown in overnight all the time. It's too much…First is what is covered under your insurance. Part of the treatment is medication, but the other part of the treatment is the nutrition. The nutrition many times is not considered medically necessary but is medically necessary…Then you are constantly battling with your insurance company. It's hard enough emotionally to deal with the condition, but then to have to be bullied by insurance companies. So many, my family included, but so many families have given up so much of their time and money. The amount of money that we spent the first five years that [she] was diagnosed, we were living in poverty, but our income was well above poverty…we could not comprehend how it could cost this much money out of our pocket to be able to keep her alive….Then your insurance company—every time you get a call, you think this is when they are going to cancel us; this is when they are going to say they are not going to pay for this anymore.”
*[12d] Provider participant*: “I've had with families that surprises me they think about this early is cost of care. I've had families who really want to know how many outpatient visits are they going to have per year. How many expected inpatient visits are they going to have per year: What are the costs of the medications: Really very financial detail… we cover such a large geographic area, so it's not uncommon for our patients to be 12 or 14 hours away each way. They're really thinking about time away from work, spending the night, and coming to see us. I've been surprised how many patients have made medical treatment decisions based on those kinds of cost.”

**Box 13: Phases of childhood and developmental milestones**

*[13a] Parent participant: *“I was talking to the dietician about green beans, and she said, ‘Well, once he's taking more than a few tablespoons at a time, I'm going to have to know how much he had so I can take some protein away from him out of the formula.’ I really thought, ‘Man, this is the easy part. Mixing formula every day and measuring, that's been easy compared to knowing specifically whats going in his mouth.’”
*[13b] Provider participant*: “The cadence of care for these kids is they have catastrophic episodes. Then they have a little honeymoon. Then they are about roughly six months to a year old when they do not have much happen to them because you control everything that goes into them. Then you start feeding them. They get a little rockier. Then they are up and down, and up and down.”
*[13c] Provider participant*: “Having to deal with unpalatable medications, how to get their kids to take them, and I think dealing with compliance for a long‐term, complex, medical condition is difficult, especially with—hard as toddlers, and then it gets a little bit easier, and then you have an adolescent who wants to have some control over their lives, and refuses to take their medicine, or says they do and do not, and things. I think those are a lot of things that our families struggle with.”
*[13d] Parent participant: *“She went to a preschool classroom two days a week, but if any of the kids were sick or if she was not feeling 100%, she was probably not there as much as half the time. She missed a lot because we just constantly had to monitor certain things.”
*[13e] Parent participant*: “The doctor made it pretty clear that she probably will not go to a normal school. Yeah, the diet poses serious challenges if she's going to be going to a school. We have not figured that problem out yet.”
*[13f] Parent participant*: “If she would've missed one of her doses of medicine before her transplant, that was a guaranteed ticket to Hopkins. As she became older, and she did not need mom and dad to shove medicine in her mouth anymore, and it became her job, for sure, I thought about that and worried about that, probably more as a teenager.”
*[13g] Parent participant*: “It's just a new chapter in our lives. In order for [my daughter] to—the possibility of her being able to maybe go to college and—I'd like for her to be able to go to college and maybe go away to college someday…just be able to think about things like that. I do not know. If we cannot get it under control now, I do not know how we are going to be able to get it under control in the next couple of years.”
*[13h] Provider participant*: “Especially with the older children, are you getting older, and who's going to take care of this—who's going to manage the child who does not have a liver transplant after you are dead and gone or if you become incapacitated: If life goes on, you never know what's going to happen around the corner. With the younger patients, I usually do not take that approach, but as the patients get into their teenage years, it's a question of, well, who is going to manage this, a sibling, and are they willing to do it: That's something that really is important to think about.”

**Table A3** Alignment between urea cycle disorders (UCD) treatment decision‐making framework concepts and previous research on decision‐making in paediatric transplant

**Table:**

Concept	Alignment with previous research
Peer‐to‐peer interaction	Dellon et al and Higgins & Kayser‐Jones both described engaging with other affected families and prior transplant recipients as an element of decision making among patients with cystic fibrosis and complex heart conditions, respectively. 26, 35 These types of peer‐to‐peer interactions are also reflected in this study's framework as a key driver of treatment choice in UCD.
Metabolic physician approach to treatment and guidance	Dellon et al, Hankins et al, and Pentz et al described trust in the recommendations of medical providers as another common factor for transplant related decision‐making in cystic fibrosis, sick cell anemia, and pediatric cancer patients. 25, 35, 41 This concept is discussed in the framework in terms of the metabolic physician's relationship with the family and the impact of their opinion and approach on the parent's perception of LT vs MM. Together with the previously published literature, study findings further support the notion that guidance from providers greatly influences treatment choice in complex, chronic pediatric conditions like UCD where transplant is a consideration.
Phases of childhood and developmental milestones	Previously published qualitative studies addressing patient and family experiences with inherited metabolic disorders identified life‐transitions as a major challenge for children and families. One such study cited problems with adherence to diet for phenylketonuria patients during adolescence. 47 Others highlighted social transitions across the lifespan as a challenge for patients diagnosed with various inherited metabolic disorders. ^48–50^ The existing literature does not explicitly discuss the role of life‐transitions in influencing treatment decisions but rather, highlights these transitions as a challenge for patients and families. Findings from this study expand on the existing literature by describing not only the inherent challenges of childhood transitions in rare disease but the ways in which developmental milestones frame treatment choice among families affected by inherited metabolic disorders like UCD.
